# Progress towards millennium development goal 1 in northern rural Nicaragua: Findings from a health and demographic surveillance site

**DOI:** 10.1186/1475-9276-11-43

**Published:** 2012-08-15

**Authors:** Wilton Pérez, Elmer Zelaya Blandón, Lars-Åke Persson, Rodolfo Peña, Carina Källestål

**Affiliations:** 1 International Maternal and Child Health (IMCH), Department of Women’s and Children’s Health Uppsala University, Uppsala, 75185, Sweden; 2 Asociación para el Desarrollo Económico y Social de El Espino (APRODESE), León, Nicaragua; 3 The Centre for Research and Interventions in Health (CIS), León, Nicaragua

**Keywords:** Poverty reduction, Transition, Rural, Interventions, Microcredit, Home gardening, Technical training, MDG 1

## Abstract

**Background:**

Millennium Development Goal 1 encourages local initiatives for the eradication of extreme poverty. However, monitoring is indispensable to insure that actions performed at higher policy levels attain success. Poverty in rural areas in low- and middle-income countries remains chronic. Nevertheless, a rural area (Cuatro Santos) in northern Nicaragua has made substantial progress toward poverty eradication by 2015. We examined the level of poverty there and described interventions aimed at reducing it.

**Methods:**

Household data collected from a Health and Demographic Surveillance System was used to analyze poverty and the transition out of it, as well as background information on family members. In the follow-up, information about specific interventions (i.e., installation of piped drinking water, latrines, access to microcredit, home gardening, and technical education) linked them to the demographic data. A propensity score was used to measure the association between the interventions and the resulting transition from poverty.

**Results:**

Between 2004 and 2009, poverty was reduced as a number of interventions increased. Although microcredit was inequitably distributed across the population, combined with home gardening and technical training, it resulted in significant poverty reduction in this rural area.

**Conclusions:**

Sustainable interventions reduced poverty in the rural areas studied by about one- third.

## Background

Strategies for the reduction of poverty are a complex and multi-faceted undertaking [1,2]. Definitions of poverty range from concepts of vulnerability, social exclusion, and deprivation to low or unsustainable development. The agenda for alleviating worldwide poverty according to the first target of the Millennium Development Goals (MDG 1) states that the number of the people living on less than $1.25 a day in low- and middle-income countries, which was 45% in 1990, must be halved by 2015 [3]. It stipulates that, beginning in 2005, the poorest nations commit to raising at least 0.4 billion people out of poverty. Although poverty reduction has been demonstrated in some countries, inadequate or lagging progress characterizes others [4-6].

Poverty is a social determinant for health [7,8]. Poor people often have too little food, unsafe water, and inadequate sanitary conditions, making them vulnerable to malnutrition, high mortality, and disease. The abject poor also face barriers in seeking access to quality health care. In addition, if a family member becomes ill, high costs are incurred and limited resources set aside for basic needs are drained [9].

In rural areas where agriculture has remained the main source of economic growth, poverty is more severe and persistent than in cities [10]. However, as educational levels increase, other income-generating activities (i.e., small businesses or employment outside the rural area) complement a family’s earnings and help reduce poverty [11-15]. With five years remaining to achieve the MDGs, there is an urgent need show that poverty reduction in rural areas can be accomplished at reasonable cost using feasible means.

The literature on poverty dynamics and the transition out of poverty refers to socioeconomic status changes that such households experience during a given time frame [16,17]. Several studies have analyzed poverty transition in order to follow trends, understand determinants, and evaluate policies and programs [18]. However, such an approach is only possible when longitudinal data are available, something that is a limiting factor in most low- and middle-income countries.

### Nicaragua and poverty

Although Nicaragua is committed to achieving the MDGs, poverty is still widespread, especially in rural areas, where 68% of the population lives in poverty or extreme poverty. This figure is five times greater than in urban areas of the country. Recent reports have shown only a small overall reduction in poverty in Nicaragua (4% from 1993 to 2005). However, a major reduction in poverty has been realized in the rural sector [19]. Nevertheless, the unequal distribution of resources continues to be a major barrier for economic growth, particularly among the poorest segment of the population [20,21].

Our study focuses on development in a low-income setting, describing pro-poor initiatives and their relation to poverty reduction in a rural community in northern Nicaragua. The study is based on panel data from the Nicaraguan Health and Demographic Surveillance Site (HDSS).

## Methods

### Study setting

**Figure 1:**
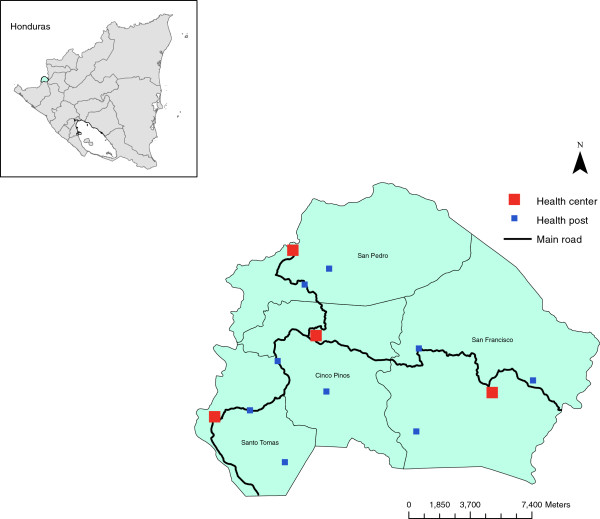
Map showing the study area, of Cuatro Santos, in Nicaragua.

The study was carried out in four municipalities that are collectively known as Cuatro Santos, situated about 250 kilometers northwest of the capital, Managua, in a mountainous 309 km^2^ area along the border with Honduras (Figure 1). Cuatro Santos has 25,000 inhabitants residing in 5,000 households. It typically has a wet season from May to November, with the highest rainfall levels in October, and a dry season from December to April. The hottest temperatures occur in March and April. In 1998, this area was heavily impacted by Hurricane Mitch, the most destructive tropical storm of that year.

Agriculture and cattle breeding are the chief economic activities in the region, and beans are the principal crop grown for consumption and marketing. In 2007–2008, the main road that connects the municipalities of Cuatro Santos was paved with funds from the international community.

### Data sources and collection methods

A population-based HDSS similar to those that serve to monitor health and demographic indicators in other low-income countries was created in Cuatro Santos [22,23]. In order to establish a population cohort, a baseline survey of households was carried out in the middle of 2004, with follow-ups in 2007 and 2009. The survey collected information on living conditions, family members, and vital events (e.g., deaths, births, and migration). Geographical locations for fieldwork were established by Global Positioning System (GPS) technology. Data quality was controlled by field supervisors and re-interviewing was done as necessary.

In 2009, information on interventions became available through an added module in the updated HDSS. Data on piped drinking water and latrine installations were collected, together with dates of installation. In addition, material was gathered on access to microcredit, home garden cultivation, and household members who had received technical training (such as courses in electrical installation, cooking, carpentry, or welding).

### Intervention design and evaluation

Our study did not examine how interventions were delivered or who provided them. Household participation in an intervention program was either self-selected or a household may have been approached by a provider who targeted a certain population. Non-governmental organizations (NGOs) and central government agencies in the area implemented similar programs, so the starting time of these projects varied. Because of this complication, the design of the interventions was considered non-randomized. The association between interventions and poverty transition was investigated with a formative evaluation design that was performed before the conclusion of the intervention in order to convey information to the local government and NGOs that might enhance a project’s effectiveness.

### Definition of variables

The household poverty measure we used was based on the Unsatisfied Basic Needs Index (UBNI) previously employed in several studies in Nicaragua [24]. This index is built on four components: 1) housing construction, 2) access to water and a latrine, 3) enrollment of children in primary school, 4) education of head of household and dependency ratio (i.e., the number of people in a family unit who are < 15 or > 65, divided by the number of people between 15 and 65). The housing component was considered inadequate (unsatisfied) if the walls of a house were built of wood, palm leaves, plastic, or cardboard, or if the house had an earthen floor. The second component was unsatisfied if water was gotten from a well, a river, or purchased in barrels; or if a house had no latrine. The third component was unsatisfied if there were children in the house between the ages of 7 and 14 who were not attending school. The last component was unsatisfied if the head of household had no formal education (i.e., was either illiterate or had not completed primary school) and the dependency ratio was greater than 2. Each unsatisfied need was scored 1 UBN; a satisfied need was scored 0.

For the purpose of our study, “non-poor households” were defined as those with less than 2 UBNs; “poor households” with 2 to 3 UBNs; and “extremely poor households” with 4 UBNs. The household poverty transition from 2004 to 2009 was defined as a change in the poverty status during that interval. Household size was defined as the number of family members residing in the household at the time of the field interview. Migration was defined as having at least one family member ages 18 to 65 who had migrated for economic reasons during the study period to a place outside the survey area (i.e., fixed or seasonal job or searching for an employment).

All intervention variables (piped drinking water, installation of latrines, home gardening, microcredit, and technical training for those ages 15 to 30) were defined as binary (1/0, either participated or did not participate).

The illiteracy rate was measured as the proportion of people > 10 years of age who did not know how to read and write. The under-5 mortality rate (U5MR) was defined as the number of deaths before a child’s fifth birthday, divided by the number of live births. The fertility rate was defined as the number of children per women of reproductive age (15 to 49 years).

### Data analysis

Household poverty was assessed in 2004 and 2009 and the number of interventions carried out in the study area was described (1990–1994, 1995–1999, 2000–2004, and 2005–2009). The rate for microcredit, home gardening, and technical training was stratified by poverty level in 2004 and 2009 using the Fleiss quadratic 95% confidence interval (CI).

A panel of households was used to analyze the poverty transition from 2004 to 2009 so that the incidence of that transition could be computed. Households that no longer could be located in 2009 or new households that did not appear in the 2004 survey were not included in the analysis.

Because data on poverty was not available in the study area before 2004, we measured the effect of the interventions upon the poverty transition between 2004 and 2009. The proportion of households who benefited from microcredits, home gardens, or technical education was analyzed by poverty transition with its respective 95% CI. We then assessed the adjusted association of interventions in relation to poverty transition. Microcredits, home gardening, and technical training were the exposures, and the outcome was the transition from poor to non-poor. The propensity score (PS) approach was applied to balance confounders among the interventions in order to reduce selection bias due to a lack of randomization in the assignment of interventions [25]. Other covariates (such as family size at baseline, sex, age of head of household, and migration) were accounted for in the model. Our rationale for including these covariates was that greater family size among those who are poor might imply fewer investments in family needs. Even when poverty is disproportionate between female and male heads of households, poverty reduction is linked to this gender issue. We also considered the age of the head of household because younger people generally have a greater capacity for work than older ones. Having a family member who migrated to another country is likely to have an impact on poverty reduction because household finances may improve through remittances. However, we did not collect information on such funds flowing back to families.

For each intervention, a PS was computed using logistic regression. Once the PS was established, the intervention per se and the PS values were included in the multivariable model, with poverty transition as the outcome variable. The c-statistic and the Brier score were used to assess the discriminatory and predictive ability of the PS model. Values for the c-statistic > 0.80 or the Brier score < 0.25 were considered good. Balance was assessed with the overlapped distribution of the PS between each intervention.

A generalized linear model of binary data for common outcomes (at least 10%) was applied [26]. The adjusted prevalence ratio was computed with its 95% CI. Stata 12.0 (Stata Corporation, College Station, Texas) was used for statistical analysis.

### Ethical consideration

The study was approved by the Medical Faculty of National University in León, Nicaragua. Verbal informed consent was obtained from all participants prior to enrollment in the study.

## Results

Table 1 presents some relevant indicators for the area studied, which in 2004 encompassed 4,451 households containing 24,095 inhabitants. By 2009 the number of households had risen to 5,037 and the population of those households increased to 25,018. Of the 4,451 households recorded in 2004, 3,852 were still present in 2009, a reduction of 599. On the other hand, 1,185 households appeared in 2009 that were not there in 2004. An average household consisted of five people, with a slightly higher ratio of females to males. Women were the heads of one-quarter of the households. The proportion of households with at least one family member who migrated increased 3-fold between 2004 and 2009, from 2% to 6%. Almost all children ages 7 to 14 were enrolled in school: enrollment increased 4% during the study period, reaching a high of 98% in 2009. The under-5 mortality rate in Cuatro Santos dropped by almost one-third between 2004 and 2008.

**Table 1:** Characteristics of study area during baseline (2004) and follow-up (2009)

	**Baseline (2004)**	**Follow-up (2009)**
	**Mean (SD**^**1**^**)**	**Mean (SD)**
Number of households	4,451	5,037
Household size	5.1 (2.5)	4.9 (2.3)
Female as head of household (95% CI^2^)	25% (23–26)	23% (21–24)
Age of head of household	46 (16)	49 (16)
Migration (95% CI)	2.2% (1.8–2.7)	6.7% (6.0–7.5)
Illiteracy (95% CI)	19% (18–20)	14% (13–14)
School enrolment^3^ (95% CI)	94% (93–94)	98% (98–99)
Sex ratio (male: female)	0.98	0.99
Women of reproductive age^4^	47% (46–48)	49% (48–50)
Children under age five (95% CI)	12% (11–12)	11% (10–11)
Under-five mortality per 1000 live births^5^ (95% CI)	24 (13–40)	16.4 (7–33)
Fertility rate	2.9	2.5

^1^SD:standard deviation.

^2^CI:confidence intervals.

^3^between 7 and 14 years of age.

^4^between 15 and 49 years of age.

^5^2008 (last reliable estimation).

### Poverty reduction and poverty transition

**Figure 2:**
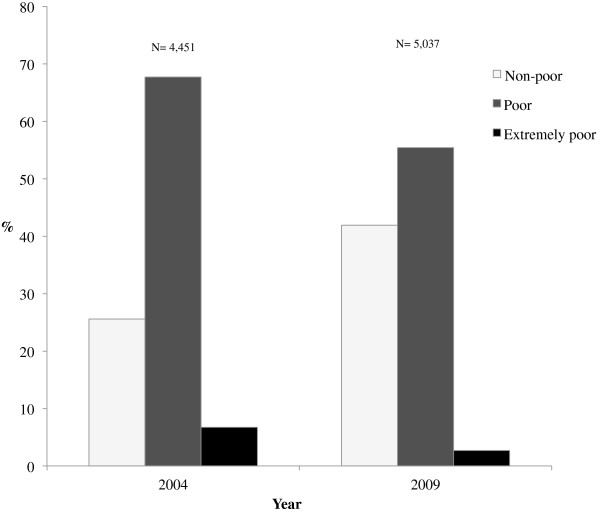
Poverty level in 2004 and 2009 in rural Cuatro Santos, Nicaragua.

Most households in Cuatro Santos were assessed as poor, followed by non-poor, and a small proportion (< 10%) that were extremely poor (Figure 2). During follow-up, the category of poor households declined by 12% and the extremely poor by 5%. Non-poor households increased by 6% during the same period. The percentage of households remaining non-poor was 83%, and 63% remained poor (Table 2). Of those households that were poor in 2004, 33% were no longer poor by 2009, and 14% of the households in extreme poverty had risen to non-poor status by the time of the follow-up.

**Table 2:** Panel of households in assessment of poverty transition from 2004 to 2009

	**Number**	**%**
Non-poor in 2004	n = 979	
Non-poor in 2009	818	83.6
Poor in 2009	160	16.3
Extremely poor in 2009	1	0.1
Poor in 2004	n = 2,574	
Non-poor in 2009	860	33.4
Poor in 2009	1,642	63.8
Extremely poor in 2009	72	2.8
Extremely-poor in 2004	n = 299	
Non-poor in 2009	41	13.7
Poor in 2009	237	79.3
Extremely poor in 2009	21	7.0
Total	3,852	100.0

### Poverty and poverty transition associated with interventions

Table 3 summarizes the interventions carried out in Cuatro Santos from 1990 to 2009. The number of people and households that benefited from household interventions (installation of piped drinking water, latrines, and the introduction of home gardening) and individual interventions (microcredits and technical training) were higher between 2005 and 2009 than during previous years.

**Table 3:** Households and individuals targeted by community interventions developed in Cuatro Santos, Nicaragua, 1990 to 2009

**Interventions**	**1990–1994**	**1995–1999**	**2000–2004**	**2005–2009**	**Total**
Households					
Piped drinking water installation	35	99	348	515	997
Latrine installation	44	480	1,089	2,331	3,944
Home gardening		6	112	737	855
Individual level					
Microcredit		9	80	1,141	1,230
Technical training	3	7	73	600	683

Table 4 shows the rate of benefiting from microcredit loans, home gardening, and technical training, as measured by poverty status in 2004 and again in 2009. In 2004, the probability of receiving any of these interventions was lower than in 2009. Significant differences were observed in the distribution of microcredit in 2009, and in the provision of technical training in both years, with the non-poor showing a higher probability of receiving these interventions than the poor or extremely poor.

**Table 4:** Distribution of microcredit, home gardens and technical training interventions rate per 1000 by level of poverty (non- poor, poor, and extremely poor), Cuatro Santos, Nicaragua, 2004 to 2009

**Program**	**2004**		**2009**	
Microcredit	Population Ages 15–65		Population Ages 15–65	
Non-poor	3,044	2.3 (7) [1.0–4.9]^1^	1,156	*83.3(174) [72.0*–*96.2]*
Poor and extremely poor	9,039	1.2 (18) [0.6–2.2]	7,274	*10.0 (125) [8.3*–*11.9]*
Home gardens	Households		Households	
Non-poor	1,139	6.1 (7) [2.6–13.2]	2,111	36.0 (76) [28.6–45.0]
Poor and extremely poor	3,356	7.6 (23) [4.4–10.4]	2,926	40.5 (117) [33.3–47.8]
Technical training	Population Ages 18–40		Population Ages 18–40	
Non-poor	1,831	*12.6 (23) [8.1*–*19.6]*	3,514	*43.3 (50) [32.5*–*57.0]*
Poor and extremely poor	5,228	*2.7 (14) [1.5*–*4.6]*	4,980	*9.5 (69) [7.4*–*12.0]*

^1^Data are presented as rate (n) with 95% CI. Italics indicate significant differences when 95% CI do not overlap.

Table 5 shows interventions between 2004 and 2009 by poverty transition groups. In the panel of poor households that rose to non-poor status, there were 216 interventions for microcredit, 143 for home gardening, and 109 for technical training. On the other hand, in the panel of poor households that remained poor or dropped to the extremely poor category, there were 324 interventions for microcredits, 197 for home gardening, and 159 for technical training. Among households that were classified as poor in 2004, the proportion of microcredits and home gardening awarded was significantly higher in those households that rose to non-poor status, compared with others which either remained poor or fell into extreme poverty by 2009 (Table 5). Significant differences by poverty transition were not evident for those receiving technical training. The proportion of non-poor households that received microcredits and maintained their non-poor status was significantly higher than those who were non-poor and fell into poverty or extreme poverty.

**Table 5:** Household distribution of microcredit, home gardens, and technical training in poverty transition groups, Cuatro Santos, Nicaragua, 2004 to 2009

**Poverty transition in household (2004 to 2009)**	**Number of households**	**Microcredit**	**Home gardening**	**Technical training**
Poor to non-poor	860	*25.1% (216) [22.2*–*28.1]*^*1*^	16.6% (143) [14.2–19.3]	12.7% (109) [10.5–15.1]
Poor to poor or extremely poor	1,714	*18.9% (324) [17.0*–*20.8]*	11.5% (197) [10.0–13.1]	9.3% (159) [7.9–10.7]
Extremely poor to non-poor	41	9.8% (4) [3.1–24.0]	7.3% (3) [1.9–21.0]	14.6% (6) [6.0–28.8]
Extremely poor to poor or extremely poor	258	19.0% (49) [14.5–24.4]	15.9% (41) [11.7–21.0]	12.4% (32) [8.7–17.2]
Non-poor to non-poor	818	*32.5% (266) [29.3*–*35.8]*	8.6% (70) [6.7–10.7]	14.8% (121) [12.4–17.4]
Non-poor to poor or extremely poor	161	*21.1% (34) [15.2*–*28.3]*	11.8% (19) [7.4–18.0]	9.3% (15) [5.4–15.1]
Total	3,852	893	473	442

^1^Data presented as percentages (n) with 95% CI. Italics indicate significant differences when 95% CI do not overlap.

Table 6 shows the association between interventions and poverty transition. The analysis revealed that microcredits (1.29; 95% CI 1.14–1.46), technical training (1.17; 95% CI 1.02–1.41), and home gardening (1.27; 95% CI 1.10–1.46) were associated with the transition from poor to non-poor by the time of the follow-up.

**Table 6:** Estimated association between interventions and poverty transition, Cuatro Santos, Nicaragua, 2004 to 2009

	**Model Poor to non-poor APR**^**1 **^**[95%CI]**	**c-statistic**	**Brier’s score for each propensity score model**
Microcredit (yes)	1.29 [1.14–1.46]	0.61	0.16
Technical training (yes)	1.20 [1.02–1.41]	0.66	0.09
Home gardening (yes)	1.27 [1.10–1.46]	0.61	0.11

^1^APR: Adjusted prevalence ratio.

## Discussion

### Main findings

The objective of achieving MDG 1 is a major challenge in many low- and middle-income countries. However, success can sometimes be attained at sufficiently low cost to facilitate putting it on a public agenda.

There has been demonstrable poverty reduction in the rural Cuatro Santos area of Nicaragua between 2004 and 2009, following an increased mix of sustainable initiatives for improved housing conditions, access to microfinance loans, and education. Our findings show that one-third of all poor and one-tenth of all extremely poor households made the transition to a better socioeconomic status during that period, suggesting that these interventions are driving transition in this setting.

### Water, sanitation, and poverty

Water and sanitation, as basic needs that are essential to life and good health, are targets of MDG 7 [27]. Their influence is strongly linked to poverty reduction [28]. Increasing the interventions we have examined has clearly diminished poverty in Cuatro Santos. Access to drinking water and sanitation is also a protective determinant for infectious diseases, especially after a natural disaster such as the hurricanes that area often experiences. Healthy family members, although they may come from poor backgrounds, are financially productive, and their resources can be reserved for needs others than medical expenses − a phenomenon recently shown in India [29].

### Microcredit, technical training and home gardening: sustainable interventions for poverty transition

Microcredit is a financial resource that helps very poor people in rural areas who need capital [30]. We found that, access to microcredit was unequally distributed, consistent with the results of studies in other settings [31]. Gaining access to a microcredit loan may require a household to have some economic resources as collateral (i.e., land, a house, savings), which few rural families might possess [32]. However, our findings show an association between microcredit and poverty alleviation in accordance with results reported elsewhere [33-35]. At the same time, it contradicts some who argue that microcredit is a catalyst for impoverishment [31].

For rural economies in transition, education is essential for poverty reduction. Since rural societies are shifting away from agriculture, technical education provides an opportunity to develop practical skills and qualify for jobs that contribute to rising out of poverty [36]. However, this kind of education was formerly only available in cities, and thus largely inaccessible for the poorest segment of the population. A center established within our study area offered several options for technical education at no cost. Although the distribution of technical education was found to be unequal, there was a significant relationship between technical education and poverty transition in this local setting.

Home gardening was also associated with the transition from poor to non-poor status. In rural areas, small-scale food cultivation is primarily used as an alternative source of food for a family, although it can also generate a small income or may provide crops that can be used to barter for other foodstuffs and household necessities [37,38].

### Strengths and limitations of the study

The first target of MDG 1 is being internationally monitored by using a poverty threshold based on the expenditure level of $1.25 per day. However, our data did not permit us to apply this calculation. Instead, we endeavored to assess poverty by using UBNs, that is an approach feasible for poor settings where multidimensional poverty overlaps. Selection bias regarding households that receive interventions may have affected our estimates and thus prevents generalization to the whole study area. For example, piped drinking water installation is the responsibility of the National Institute of Water in Nicaragua, supported by local NGOs. However, the sites where these pipes are installed are not equally distributed geographically, that is, some NGOs will only assist households that are on or near main roads where heavy machinery for drilling and running pipelines has easier access. Moreover, water installation requires electrical power, which in most remote communities is inadequate or non-existent [39].

Recourse to microcredit is probably easier for those who have previously received such a loan. However, we do not believe there is a large selection bias involved here because households that had been given multiple loans were rare (3%). Selection bias appears to be greater in the case of technical training, as people in remote areas did not have access to these programs.

The HDSS was unable to collect temporal (short duration) migrations occurring at least two months before each updated census, which may have affected the association between interventions and poverty transition. Our results are only representative of the northern rural areas of Nicaragua, excluding the rural region of the Caribbean.

Head of household was self-reported in the HDSS census. In rural areas where *machismo* (masculine pride) is dominant, a respondent might answer in the name of an absent male or a male who is not the person responsible for making household financial decisions. Therefore, the definition of head of household may likely suffer from misclassification, although the proportion of females who were heads of households was consistent with that found in rural areas of Latin American [40]. Because the present study was not randomized, it is possible that some unknown variable influenced the outcome. However, we employed the PS approach that takes into account the lack of randomization with regard to interventions. We are aware of the need to include in future work other unobservable covariates that are also related to poverty transition in rural areas.

## Conclusions

We have considered some initiatives for poverty reduction, but poverty is only one of several dimensions of human development. Another line of exploration would be to measure the impact that roads could have for economic development in impoverished areas, especially when programs are scaled-up with spatial preferences to families living close to roads. It might also be fruitful to study the capacity of female heads of households to lift rural families out of poverty. This has been shown in other settings and may require broadening the definition of head of household [41].

Rural poverty can be reduced by sustainable interventions. The poorest people in rural areas are eager to participate in initiatives that improve their life. If the example of such development in Nicaragua can be implemented in other parts of the world, it may be encouraging step forward in the ongoing struggle to reduce impoverishment.

## Competing interests

The authors declare that they have no competing interests.

## Authors’ contributions

LÅP, CK, RP, and EZ designed the HDSS. EZ and RP supervised data collection. CK designed the intervention survey. WP performed the statistical analysis and wrote the manuscript. EZ, CK, RP, and LÅP participated in data interpretation and drafting of the manuscript. All authors read and approved the final manuscript.
