# Correction: Progress towards millennium development goal 1 in northern rural Nicaragua: Findings from a health and demographic surveillance site

**DOI:** 10.1186/1475-9276-11-72

**Published:** 2012-11-23

**Authors:** Wilton Pérez, Elmer Zelaya Blandón, Lars-Åke Persson, Rodolfo Peña, Carina Källestål

**Affiliations:** 1 International Maternal and Child Health (IMCH), Department of Women’s and Children’s Health, Uppsala University, Uppsala, 75185, Sweden; 2 Asociación para el Desarrollo Económico y Social de El Espino (APRODESE), León, Nicaragua; 3 The Centre for Research and Interventions in Health (CIS), León, Nicaragua

## Correction

After publication of our article Pérez et al Int J Equity Health 2012 11:43, we became aware of some errors in the final published version, for which the corresponding author accepts full responsibility.

Page 3: third paragraph, Lines 2–4: “…, microcredit, and technical training for those ages 15 to 30” should be read “…, microcredit for those ages 15 to 65, and technical training for those ages 18 to 40”.

The original Table four (Table 1) has some incorrect figures. We have corrected these figures in the updated table.

**Table 1:** Distribution of microcredit, home gardens and technical training interventions rate per 1000 by level of poverty (non-poor, poor, and extremely poor), Cuatro Santos, Nicaragua, 2004 to 2009

**Program**	**2004**		**2009**	
Microcredit	Population Ages 15–65		Population Ages 15–65	
Non-poor	3,044	2.3 (7) [1.0–4.9]^1^	6,229	*27.9 (174) [24.0–32.4]*
Poor and extremely poor	9,039	1.2 (11) [0.6–2.2]	8,439	*14.8 (125) [12.3–17.6]*
Home gardens	Households		Households	
Non-poor	1,095	6.4 (7) [2.8–13.7]	2,111	36.0 (76) [28.6–45.0]
Poor and extremely poor	3,356	6.9 (23) [4.4–10.4]	2,926	40.0 (117) [33.3–47.8]
Technical training	Population Ages 18–40		Population Ages 18–40	
Non-poor	1,831	*12.6 (23) [8.1–19.1]*	3,514	14.2 (50) [10.6–18.8]
Poor and extremely poor	5,228	*2.7 (14) [1.5–4.6]*	4,980	13.8 (69) [10.8–17.5]

^1^Data are presented as rate (n) with 95% CI. Italics indicate significant differences when 95% CI do not overlap.

Because new corrected figures in the original Table four (Table 1) the following statement should be changed: Page 5: third paragraph, Line 7: “…provision of technical training in both years,…” should be read “…provision of technical training in 2004,…”. Page 7: first paragraph, Lines 7–8: “Although the distribution of technical education was found to be unequal, there was a significant relationship between…” should be read “Although the distribution of technical education was found to be unequal in 2004, it was equal in 2009, and it showed a significant relationship between…”. These corrections do not affect the rest of the data analysis.

We regret any inconvenience that this oversight may have caused.
